# The value of ^18^F-FDG PET before and after induction chemotherapy for the early prediction of a poor pathologic response to subsequent preoperative chemoradiotherapy in oesophageal adenocarcinoma

**DOI:** 10.1007/s00259-016-3478-2

**Published:** 2016-08-11

**Authors:** Peter S. N. van Rossum, David V. Fried, Lifei Zhang, Wayne L. Hofstetter, Linus Ho, Gert J. Meijer, Brett W. Carter, Laurence E. Court, Steven H. Lin

**Affiliations:** 1 Department of Radiation Oncology, The University of Texas MD Anderson Cancer Center, 1515 Holcombe Blvd, Houston, TX 77030 USA; 2 Department of Radiation Oncology, University Medical Center Utrecht, PO Box 85500, Q00.3.11, 3508GA Utrecht, The Netherlands; 3 Department of Radiation Physics, The University of Texas MD Anderson Cancer Center, Houston, TX USA; 4 Department of Thoracic and Cardiovascular Surgery, The University of Texas MD Anderson Cancer Center, Houston, TX USA; 5 Department of Gastrointestinal Medical Oncology, The University of Texas MD Anderson Cancer Center, Houston, TX USA; 6 Department of Diagnostic Radiology, The University of Texas MD Anderson Cancer Center, Houston, TX USA

**Keywords:** ^18^F-FDG PET, Treatment response, Oesophageal cancer, Chemotherapy, Chemoradiotherapy

## Abstract

**Purpose:**

The purpose of our study was to determine the value of ^18^F-FDG PET before and after induction chemotherapy in patients with oesophageal adenocarcinoma for the early prediction of a poor pathologic response to subsequent preoperative chemoradiotherapy (CRT).

**Methods:**

In 70 consecutive patients receiving a three-step treatment strategy of induction chemotherapy and preoperative chemoradiotherapy for oesophageal adenocarcinoma, ^18^F-FDG PET scans were performed before and after induction chemotherapy (before preoperative CRT). SUV_max_, SUV_mean_, metabolic tumour volume (MTV), and total lesion glycolysis (TLG) were determined at these two time points. The predictive potential of (the change in) these parameters for a poor pathologic response, progression-free survival (PFS) and overall survival (OS) was assessed.

**Results:**

A poor pathologic response after induction chemotherapy and preoperative CRT was found in 27 patients (39 %). Patients with a poor pathologic response experienced less of a reduction in TLG after induction chemotherapy (*p* < 0.01). The change in TLG was predictive for a poor pathologic response at a threshold of −26 % (sensitivity 67 %, specificity 84 %, accuracy 77 %, PPV 72 %, NPV 80 %), yielding an area-under-the-curve of 0.74 in ROC analysis. Also, patients with a decrease in TLG lower than 26 % had a significantly worse PFS (*p* = 0.02), but not OS (*p* = 0.18).

**Conclusions:**

^18^F-FDG PET appears useful to predict a poor pathologic response as well as PFS early after induction chemotherapy in patients with oesophageal adenocarcinoma undergoing a three-step treatment strategy. As such, the early ^18^F-FDG PET response after induction chemotherapy could aid in individualizing treatment by modification or withdrawal of subsequent preoperative CRT in poor responders.

## Introduction

The long-term survival of patients with locoregionally advanced oesophageal cancer remains quite poor despite considerable advances in surgery, radiotherapy, and chemotherapy, with 5-year survival rates still below 50 % [1, 2]. Multimodality treatment strategies have been implemented in an effort to improve the outcome achieved with surgery alone [3]. Since early studies showed that adjuvant therapy did not improve outcomes [4–7], contemporary research mainly focused on neoadjuvant strategies, which resulted in improved resection rates, pathologic downstaging, and a reduction in disease recurrences [3]. As a result, preoperative concurrent chemoradiotherapy (CRT) followed by oesophagectomy is commonly applied in clinical practice [8].

An important observation in patients treated with trimodality therapy (i.e., preoperative CRT followed by oesophagectomy) is that the most common pattern of treatment failure is now distant progression [8, 9]. In an attempt to eliminate micrometastases and thereby improve the distant failure rate and overall outcome, additional induction chemotherapy before trimodality therapy has been investigated in the United States and Europe, as well as in Asia [10–29]. Results of comparative studies have been inconclusive with some studies reporting a benefit of induction chemotherapy [15, 16], while others were equivocal [27, 29]. Nonetheless, induction chemotherapy is thought to have a number of potential advantages including improvement of swallowing/nutritional status and obviating the need for feeding tubes in patients presenting with dysphagia [11, 12, 14, 18, 19, 22, 24]. More importantly, it has been suggested that the use of induction chemotherapy may permit early identification of poorly responding patients in whom neoadjuvant treatment is ineffective or even harmful [24, 30–32].

^18^F-fluorodeoxyglucose positron emission tomography (^18^F-FDG PET) is a well-established imaging modality for initial staging and re-staging after preoperative CRT for the detection of distant (interval) metastases [33–37]. ^18^F-FDG PET has been shown to be more accurate than other modalities in predicting pathologic response to neoadjuvant chemotherapy or CRT for oesophageal cancer [38, 39]. However, current evidence is limited with regard to the value of ^18^F-FDG PET for response prediction in the setting of a three-step strategy of induction chemotherapy and preoperative CRT followed by oesophagectomy. Therefore, the aim of this study was to determine the value of ^18^F-FDG PET scanning at baseline and after induction chemotherapy for the early prediction of a poor versus good pathologic response (i.e. >10 % versus ≤10 % residual carcinoma) to subsequent preoperative CRT.

## Material and methods

This retrospective study has been approved by our Institutional Review Board, and the need for written informed consent was waived. The study was conducted in accordance with the Health Insurance Portability and Accountability Act (HIPAA) and the checklist from the STAndards for the Reporting of Diagnostic accuracy studies (STARD) statement (http://www.stard-statement.org) [40].

### Study population

From a prospectively acquired database, we extracted all consecutive patients with a biopsy-proven potentially resectable adenocarcinoma of the oesophagus or gastro-oesophageal junction and no distant metastases that underwent a three-step treatment strategy of induction chemotherapy and preoperative chemoradiotherapy followed by surgery at our institution from March 2006 to February 2013. Patients were excluded if one of two ^18^F-FDG PET scans of interest were either not available or acquired at another institution. Also, non-FDG-avid tumours at baseline, Siewert type 3 gastro-oesophageal junction tumours, and patients with a stent in-situ at the time of scanning were excluded. Finally, patients with a time interval between completion of preoperative chemoradiation and surgery of less than 5 weeks or more than 14 weeks - indicating urgent and salvage resections, respectively - were excluded.

### Treatment regimen

All patients were treated by induction chemotherapy and subsequent external beam radiation with concurrent chemotherapy. The backbone of induction chemotherapy generally consisted of a fluoropyrimidine (intravenous 5-FU or oral capecitabine) and oxaliplatin, with the addition of either leucovorin (54 % of cases) or docetaxel (37 % of cases) [17, 27]. Other (sporadic) induction chemotherapy regimens included carboplatin/paclitaxel (3 %), cisplatin/paclitaxel (1.5 %), cisplatin/irinotecan (1.5 %), 5-FU monotherapy (1.5 %) and capecitabine/oxaliplatin/epirubicin (1.5 %). Radiation therapy consisted of a total radiation dose of 45.0 Gy (4 %) or 50.4 Gy (96 %) delivered in daily fractions of 1.8 Gy using intensity modulated radiation therapy (IMRT; 69 %) or proton therapy (31 %). The chemotherapy concurrently administered with radiation generally consisted of a fluoropyrimidine (intravenous or oral) with either a platinum compound (69 %) or docetaxel (17 %). Other (sporadic) concurrent chemotherapy regimens included carboplatin/paclitaxel (3 %), 5-FU/paclitaxel (3 %), 5-FU/oxaliplatin/docetaxel (3 %), oxaliplatin/docetaxel/irinotecan (3 %), oxaliplatin/docetaxel (1 %), and cisplatin/irinotecan (1 %). After completion of chemoradiation, either a transthoracic (Ivor-Lewis), transhiatal, total (three-field technique), or minimally invasive oesophagectomy was performed with curative intent at the discretion of the treating surgeon.

### Histopathologic assessment

Histopathologic examination of the resected specimen was standardized in accordance with the seventh edition of the American Joint Committee on Cancer protocol for TNM-classification [41]. The degree of pathologic response to neoadjuvant treatment was graded as follows [42]: complete absence of residual cancer (tumour regression grade [TRG] 1), 1-10 % residual carcinoma (TRG 2), 11-50 % residual carcinoma (TRG 3), and >50 % residual carcinoma (TRG 4). A poor pathologic response (defined as TRG 3–4) as opposed to a good pathologic response (defined as TRG 1–2) was considered the reference standard of this study.

### Image acquisition

^18^F-FDG PET/computed tomography (CT) scans were performed on an integrated PET/CT system (Discovery RX, ST, or STE; GE Medical Systems, Milwaukee [WI], USA). Before ^18^F-FDG PET, a CT scan was acquired (120 kV peaks, 300 mA, 0.5 seconds rotation, pitch of 1.375, slice thickness 3.75 mm, and slice interval 3.27 mm) for attenuation correction purposes. ^18^F-FDG PET scans were acquired 60–90 minutes after administration of ^18^F FDG with a dose of 555–740 MBq, in either two-dimensional (2-D) or three-dimensional (3-D) acquisition mode at 3–5 minutes per bed position. Images were reconstructed using ordered-subset expectation maximization in 2-D or iterative reconstruction in 3-D images. All analyses were performed on the attenuation-corrected images.

### Image analysis

The primary tumour was defined as the volume of interest (VOI) and delineated on the ^18^F-FDG PET scans using a semi-automatic gradient-based delineation method from commercially available software (MIM Software, Cleveland [OH], USA). This contouring method has recently been validated in a multi-observer study that showed superiority over manual and threshold methods [43]. The following quantitative features were extracted from the VOIs of the ^18^F-FDG PET scans at baseline and after induction chemotherapy (before preoperative CRT): maximum and mean standardized uptake value (SUV_max_ and SUV_mean_), metabolic tumour volume (MTV) and total lesion glycolysis (TLG). The MTV was automatically calculated by the software by summing up the areas within each two-dimensional transverse tumour contour multiplied by the corresponding slice thickness. The TLG was calculated by multiplying MTV by SUV_mean_ [44]. In addition, the relative changes (in %) of these parameters between ^18^F-FDG PET at baseline and ^18^F-FDG PET after induction chemotherapy were calculated and included in the analysis.

### Statistical analysis

First, the association between clinical parameters and poor versus good pathologic response was studied using the chi-square test (or Fisher’s exact test in case of small cell count) for categorical parameters, and Student’s T-test for parametric continuous parameters. The association between the quantitative ^18^F-FDG PET parameters and pathologic response was quantified using logistic regression analysis providing odds ratios (ORs) with 95 % confidence intervals (CIs). Multiple ^18^F-FDG PET parameters were logarithmically transformed to meet the assumption of linearity on the logit scale. For these parameters, the relative changes (%) were calculated using the logarithmically transformed parameter values before and after induction chemotherapy.

Second, receiver operating characteristics (ROC) curve analyses (providing area-under-the-curve [AUC] values) were used to assess the potential of the studied ^18^F-FDG PET parameters to discriminate poor responders from good responders. For the ^18^F-FDG PET parameter with the highest discriminatory ability (AUC), the sensitivity, specificity, accuracy, positive predictive value (PPV), and negative predictive value (NPV) were calculated for an optimal threshold that was determined by giving equal weight to sensitivity and specificity on the ROC curve.

Third, the Kaplan-Meier method was applied to estimate progression-free and overall survival differences among patients predicted to have a poor versus good response based on the ^18^F-FDG PET parameter with the highest discriminatory ability. For the survival analysis, the log-rank test was used to determine significance. Progression-free survival and overall survival were calculated from the starting date of induction chemotherapy to the date of disease progression after surgery or the date of death, respectively. In patients who were free of disease progression or alive at last follow-up, the date of last follow-up was used to censor progression-free or overall survival times, respectively. Statistical analysis was performed using SPSS 23.0 (IBM Corp., Armonk [NY], USA) and R 3.1.2 open-source software (http://www.R-project.org). A p-value <0.05 was considered statistically significant.

## Results

From a total of 132 patients with an oesophageal adenocarcinoma who underwent induction chemotherapy and preoperative chemoradiotherapy followed by surgery in the study period, 70 were considered eligible for analysis. Some excluded patients missed at least one of two ^18^F-FDG PET scans of interest performed at our institution (*n* = 28); these patients had similar response and survival rates compared to the included cohort. Other excluded patients had a Siewert type 3 gastro-oesophageal junction tumour (*n* = 15), a non-FDG avid tumour (*n* = 6), a stent in-situ at the time of scanning (*n* = 1), or underwent an urgent or salvage oesophagectomy (*n* = 1 and *n* = 11, respectively).

Among the 70 eligible patients, 27 (39 %) had a poor pathologic response (TRG 3–4) to neoadjuvant treatment, whereas 43 (61 %) had a good pathologic response (TRG 1–2). Patients with a poor response had a mean age of 60 years and 96 % (*n* = 26) of them were male, whereas patients with a good response had a mean age of 59 years and 88 % (*n* = 38) of them were male. None of the studied baseline characteristics were significantly related to the pathologic response to neoadjuvant treatment (Table 1). More specifically, only small non-significant differences regarding pathologic response for the various induction chemotherapy regimens, radiation therapy characteristics and concurrent chemotherapy regimens were found. However, worse tumour characteristics (i.e., higher clinical T-stage, signet ring cell adenocarcinoma, poor differentiation grade) and co-morbidities (i.e., cardiac co-morbidity, diabetes mellitus, chronic obstructive pulmonary disease, and smoking at diagnosis) were consistently observed more frequently in the poor response group.

**Table 1:** Patient and treatment-related characteristics

Characteristic	Poor response (*n* = 27)	Good response (*n* = 43)	*p* value
Male gender	26 (96.3)	38 (88.4)	0.39
Age (years)^†^	59.9 ± 11.5	59.4 ± 10.6	0.86
BMI (kg/m^2^)^†^	30.3 ± 5.0	30.0 ± 5.0	0.79
Cardiac co-morbidity	7 (25.9)	7 (16.3)	0.33
Diabetes mellitus	7 (25.9)	8 (18.6)	0.47
COPD	3 (11.1)	1 ( 2.3)	0.29
Smoking at diagnosis	8 (29.6)	8 (18.6)	0.29
Karnofsky performance status^†^	85.6 ± 6.4	85.6 ± 6.3	0.99
Tumor location			0.70
Distal third of esophagus	25 (92.6)	38 (88.4)	
Gastro-esophageal junction	2 ( 7.4)	5 (11.6)	
EUS-based tumor length (cm)^†^	6.3 ± 2.8	6.1 ± 2.9	0.87
Histologic differentiation grade			0.35
Moderate	12 (44.4)	24 (55.8)	
Poor	15 (55.6)	19 (44.2)	
Signet ring cell adenocarcinoma	6 (22.2)	4 ( 9.3)	0.17
Clinical T-stage			0.14
cT2	1 ( 3.7)	7 (16.3)	
cT3	26 (96.3)	36 (83.7)	
Clinical N-stage			0.73
cN0	7 (26.9)	10 (23.3)	
cN+	19 (73.1)	33 (76.7)	
*Missing*	1	-	
Induction chemotherapy regimen			0.81
Fluoropyrimidine/oxaliplatin/leucovorin	14 (51.9)	24 (55.8)	
Fluoropyrimidine/oxaliplatin/docetaxel	10 (37.0)	16 (37.2)	
Other	3 (11.1)	3 ( 7.0)	
Radiation treatment modality			0.79
IMRT	18 (66.7)	30 (69.8)	
Proton therapy	9 (33.3)	13 (30.2)	
Total radiation dose			1.00
45.0 Gy	1 ( 3.7)	2 ( 4.7)	
50.4 Gy	26 (96.3)	41 (95.3)	
Concurrent chemotherapy regimen			0.25
Fluoropyrimidine/platinum	18 (66.7)	30 (69.8)	
Fluoropyrimidine/docetaxel	3 (11.1)	9 (20.9)	
Other	6 (22.2)	4 ( 9.3)	

Data are presented as numbers with percentages in parentheses

^†^: Expressed as mean ± SD.

COPD: Chronic obstructive pulmonary disease. EUS: Endoscopic ultrasound. IMRT: Intensity-modulated radiotherapy

Baseline ^18^F-FDG PET parameters, SUV_max_, and SUV_mean_ after induction chemotherapy were not related to pathologic poor versus good response (Table 2). However, both a larger MTV and a larger TLG after induction chemotherapy were significantly related to a higher chance of a poor pathologic response (*p* = 0.01). The relative changes after induction chemotherapy in ^18^F-FDG PET intensity parameters (i.e., ∆SUV_max_ and ∆SUV_mean_) and metabolic tumour volume (i.e., ∆MTV) were also significantly related to pathologic response (*p* = 0.01), and their discriminatory ability appeared to be superior compared with single time point measurements (AUC range 0.71-0.72 vs. 0.52-0.69; Table 2). The association of the relative change in (the logarithmically transformed) total lesion glycolysis (∆TLG) with pathologic response was highly significant (*p* < 0.01) and this parameter yielded the highest discriminatory ability (AUC 0.74).

**Table 2:** Logistic regression and ROC curve analysis of ^18^F-FDG PET parameters before and after induction chemotherapy for predicting poor pathologic response to chemoradiotherapy

Parameter	Poor response (*n* = 27) Median [IQR]	Good response (*n* = 43) Median [IQR]	OR	95 % CI	*p* value	AUC
^18^F-FDG PET before induction chemotherapy						
SUV_max_^†^	14.2 [ 8.2, 18.6]	14.7 [ 9.7, 20.4]	0.90	0.39 – 2.06	0.80	0.52
SUV_mean_^†^	6.5 [ 4.7, 9.4]	6.4 [ 4.9, 8.9]	0.76	0.28 – 2.08	0.60	0.53
MTV (mL)^†^	24.2 [ 14.9, 46.8]	26.4 [ 14.4, 45.1]	0.96	0.53 – 1.73	0.90	0.52
TLG^†^	171 [ 88.9, 299]	203 [ 61.1, 454]	0.93	0.61 – 1.43	0.75	0.52
^18^F-FDG PET after induction chemotherapy						
SUV_max_^†^	7.0 [ 5.2, 9.2]	5.0 [ 3.3, 7.8]	2.51	0.96 – 6.59	0.06	0.66
SUV_mean_^†^	4.3 [ 3.5, 5.2]	3.7 [ 2.6, 4.9]	2.44	0.68 – 8.77	0.17	0.62
MTV (mL)^†^	13.1 [ 7.4, 18.6]	7.0 [ 2.5, 12.0]	2.60	1.32 – 5.11	0.01*	0.69
TLG^†^	48.9 [ 28.4, 81.0]	24.5 [ 8.6, 64.7]	1.90	1.16 – 3.10	0.01*	0.68
Relative difference						
∆SUV_max_ (%)	−20.5 [−32.0,-12.5]	−32.4 [−48.8,-24.7]	1.05	1.01 – 1.09	0.01*	0.71
∆SUV_mean_ (%)	−21.3 [−32.9,-21.3]	−31.4 [−45.5,-19.7]	1.04	1.01 – 1.07	0.01*	0.71
∆MTV (%)	−13.2 [−37.2, −7.9]	−39.1 [−63.8,-26.1]	1.04	1.01 – 1.06	0.01*	0.72
∆TLG (%)	−19.5 [−34.1, −9.8]	−34.0 [−52.7,-29.9]	1.05	1.02 – 1.09	<0.01*	0.74

^†^: Logarithmically transformed for logistic regression analysis to meet the assumption of linearity on the logit scale.

*: Significantly associated with poor versus good pathologic response (*p* < 0.05).

AUC: Area under the (receiver operating characteristic [ROC]) curve. IQR: Interquartile range. MTV: Metabolic tumor volume. SUV: Standardized uptake value. TLG: Total lesion glycolysis.

**Fig. 1:**
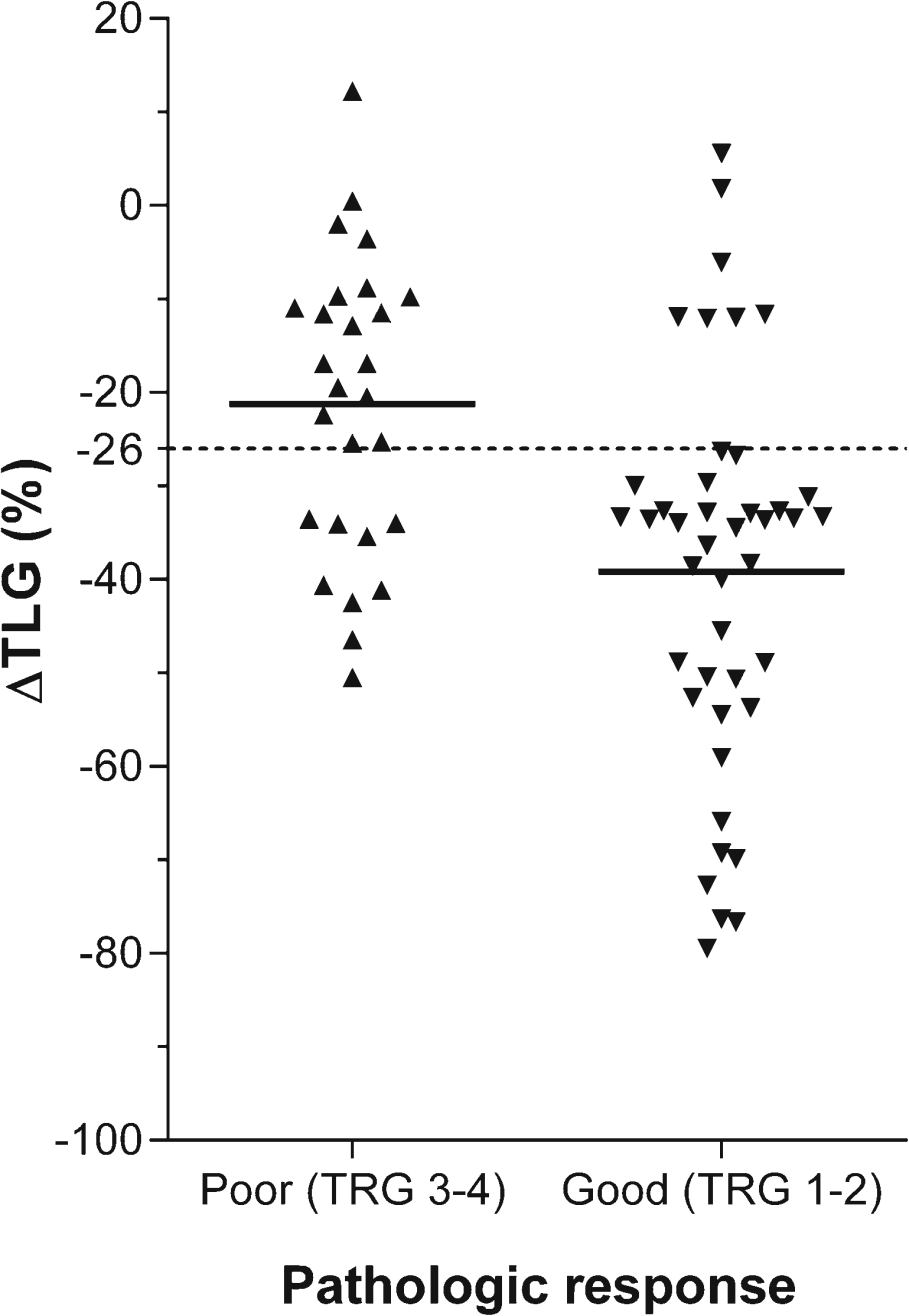
Scatter plot demonstrating the percentage of change in the logarithmically transformed total lesion glycolysis (∆TLG) after induction chemotherapy before preoperative chemoradiotherapy for oesophageal cancer in 27 poor versus 43 good pathologic responders. Horizontal continuous lines represent group means and the dotted line represents the optimal discriminatory cut-off level for ∆TLG of −26 %

The ideal cut-off value for ∆TLG to distinguish poor pathologic responders from good responders was statistically determined at −26 % (i.e., a 26 % decrease). Patients with a ∆TLG above (*n* = 25) versus belo*w* (n = 45) this threshold had a poor pathologic response in 72 % versus 20 % of cases, respectively. At the threshold of −26 %, the ∆TLG yielded a sensitivity of 67 % (95 % CI: 51-79 %), specificity of 84 % (95 % CI: 74-91 %), accuracy of 77 % (95 % CI: 65-86 %), PPV of 72 % (95 % CI: 55-85 %), and NPV of 80 % (95 % CI: 71-87 %) for predicting a poor pathologic response (Fig. 1). Of note, the threshold for the relative change in the logarithmically transformed TLG values of −26 % compared best to a threshold for the relative change in the originally scaled TLG values of −74 %. However, this originally scaled ∆TLG yielded a slightly lower predictive performance (AUC 0.71, with sensitivity 70 % [95 % CI: 54-83 %], specificity 74 % [95 % CI: 64-83 %], accuracy 73 % [95 % CI: 60-83 %], PPV 63 % [95 % CI: 49-75 %], and NPV 80 % [95 % CI: 69-89 %]).

**Fig. 2:**
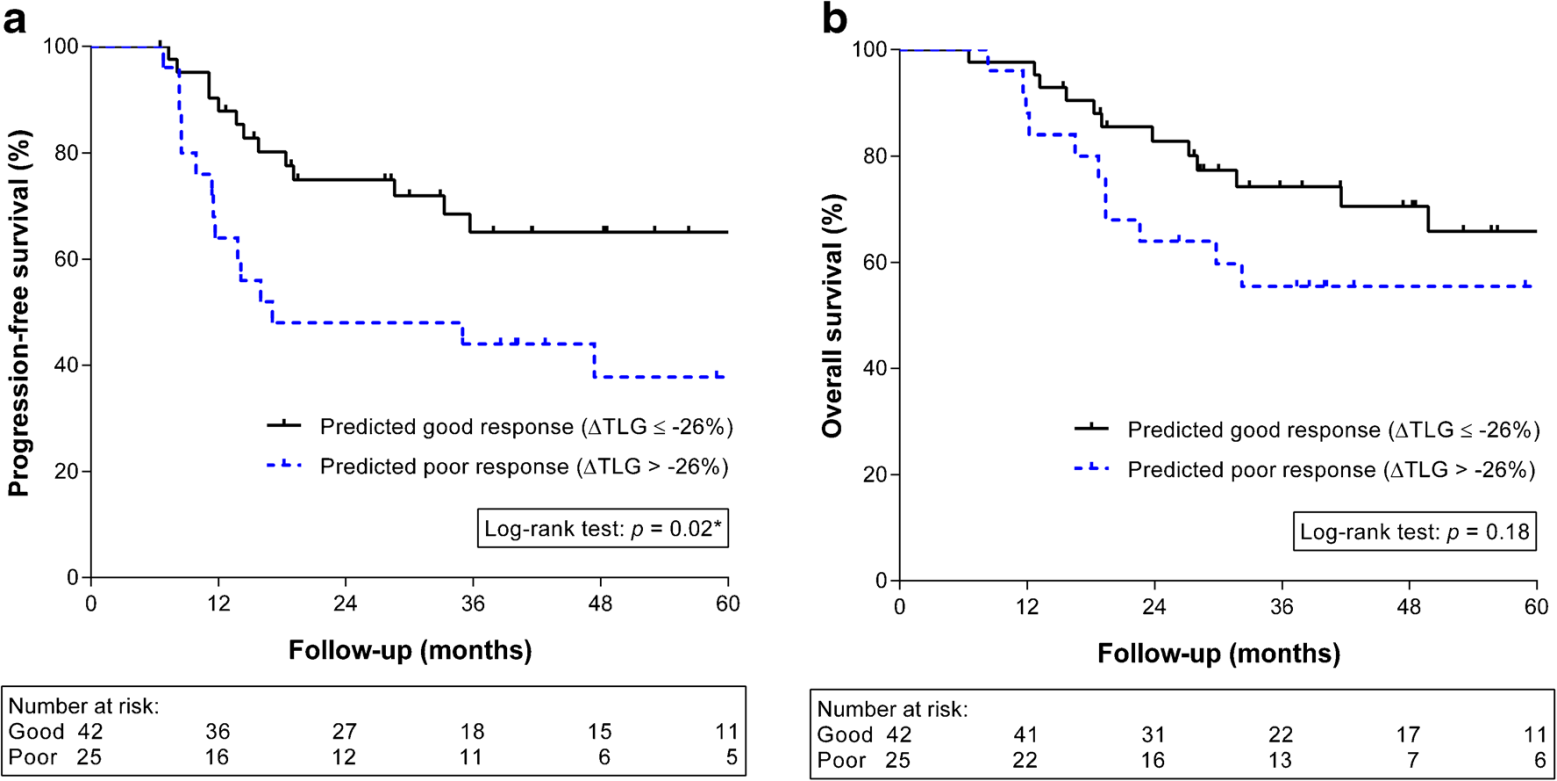
Kaplan-Meier analysis for progression-free survival (**a**) and overall survival (**b**) according to predicted good versus poor response by the change in the logarithmically transformed total lesion glycolysis (∆TLG) after induction chemotherapy before preoperative chemoradiotherapy for oesophageal cancer

Post-operative 30-day and 90-day mortality rates were 1 % (1 of 70) and 4 % (3 of 70), respectively. These three patients (who were part of the predicted good responders group) were excluded from survival analysis. For patients alive at last follow-up, the median follow-up duration was 48 months (range 15 to 99). In the 25 patients with a predicted poor response based on (the logarithmically transformed) ∆TLG the median progression-free survival was 17 months, whereas the median progression-free survival in the 42 patients with a predicted good response was not reached (Fig. 2a). The progression-free survival was significantly better for the predicted good responders compared to the predicted poor responders based on ∆TLG (*p* = 0.02). Although overall survival rates appeared higher in patients with a predicted good response (median, not reached) compared to predicted poor responders (median, 70 months), this difference was not statistically significant (*p* = 0.18; Fig. 2b).

## Discussion

In this study, the value of ^18^F-FDG PET before and after induction chemotherapy for the prediction of response to neoadjuvant treatment was investigated in patients undergoing induction chemotherapy followed by trimodality therapy for oesophageal adenocarcinoma. Significant associations were found between treatment-induced changes in studied ^18^F-FDG PET parameters and histopathologic tumour regression defined as poor response (TRG 3–4) versus good response (TRG 1–2).

A decrease of less than 26 % in (the logarithmically transformed) TLG after induction chemotherapy, indicating only a mild reduction in intensity and volume of FDG-uptake of the primary tumour, predicted a poor pathologic response with a specificity of 84 % and PPV of 72 %. This implies that the baseline (a priori) chance of a poor pathologic response of 39 % (i.e., the overall prevalence) almost doubled to 72 % (i.e., the PPV) in predicted poor responders. This is particularly interesting when considering modification of the chemotherapy regimen administered concurrently with preoperative CRT after induction chemotherapy (e.g., in patients with burdening toxicity from induction chemotherapy) or even omission of ineffective and toxic preoperative CRT in predicted poor responders. On the other hand, a strong reduction of more than 26 % in TLG after induction chemotherapy predicted a good pathologic response with a sensitivity of 67 % and NPV of 80 %. This implies that the baseline (a priori) chance of a good pathologic response of 61 % (i.e., the overall prevalence) increased to 80 % (i.e., the NPV) in predicted good responders. This indicates that ^18^F-FDG PET before and after induction chemotherapy provides a reasonable basis to encourage good responders to have induction chemotherapy and to proceed with preoperative chemoradiotherapy.

Several single-arm phase I-II studies [10–14, 19, 21–23, 25] and two retrospective comparative studies [15, 16] found promising results with the three-step treatment strategy compared to preoperative CRT without induction chemotherapy in terms of treatment response, R0 resection rates, and survival rates. However, this potential superiority was not found in a retrospective comparative study [17] and two prospective randomized phase II studies [27, 29]. One study suggested that only patients with stage III and IVa (and not stage II) disease who received induction chemotherapy had a significant survival advantage over preoperative CRT alone [16]. The three-step approach has not been evaluated in the context of a phase III trial. Therefore, the use of induction chemotherapy to improve oncologic outcomes remains a subject of debate. Nonetheless, the response to induction chemotherapy may serve as a marker for tumour sensitivity indicating whether benefit is to be expected from subsequent CRT or whether different chemotherapeutic agents should be incorporated into the preoperative CRT [24–26].

Since oesophageal cancer patients with a poor pathologic response to neoadjuvant treatment do not seem to benefit from this treatment but are exposed to its treatment-related toxicity [11, 13, 30, 31], accurately predicting pathologic response before or early during treatment would produce much-needed knowledge to help individualize therapy. In this regard, the predictive value of ^18^F-FDG PET response has previously been reported in preoperative chemotherapy studies of patients with oesophageal adenocarcinoma [45, 46]. In the subsequent MUNICON trial from that group [32], ^18^F-FDG PET-based poor responders early during preoperative chemotherapy were referred for immediate surgery rather than continuation of preoperative chemotherapy, and this discontinuation of ineffective chemotherapy did not adversely affect outcome compared with continuing such therapy [32].

The current study demonstrates that ^18^F-FDG PET before and after induction chemotherapy yields a moderate ability to predict a poor pathologic response to subsequent preoperative CRT. The value of ^18^F-FDG PET in this setting has been previously described in four smaller cohorts [20, 24, 26, 47], one of which had no histopathologic reference as no surgery was performed [26]. Similar to the current study, three previous studies with 45, 55, and 46 patients, respectively [20, 24, 47], performed ^18^F-FDG PET before and after induction chemotherapy and reported a significant association between early ^18^F-FDG PET response and histopathologic tumour regression. Two studies reported the predictive performance of ^18^F-FDG PET for predicting a poor pathologic response with sensitivities of 52 % and 68 %, and specificities of 60 % and 52 % [20, 47]. The differences with the current study (sensitivity 67 %, specificity 84 %) may be explained by varying ^18^F-FDG PET hardware, scan protocols, and reconstruction algorithms between studies [20, 47] and within one multicenter study [47], by the different applied thresholds for ^18^F-FDG PET response [20, 47], and by the different treatment regimens used in other studies [20, 47]. One previous study only reported on the value of ^18^F-FDG PET before and after induction chemotherapy to predict residual cancer as opposed to a pathologic complete response (i.e., TRG 2–4 vs. 1), and found a sensitivity of 61 % and specificity of 89 % [24]. These results led investigators to examine the use of ^18^F-FDG PET to direct preoperative therapy in patients with oesophageal cancer in the Cancer and Leukemia Group B trial 80803, which was opened in 2011 [24]. Results of that trial, in which the chemotherapy regimen to be used during preoperative CRT will be selected by ^18^F-FDG PET response after induction chemotherapy, are currently awaited.

Although ^18^F-FDG PET before and after induction chemotherapy appears to have a reasonable discriminatory ability for predicting pathologic response, it remains suboptimal. Studies have been focusing mainly on quantitative parameters, but subjective assessment by clinicians is thought to have some additional potential, as it is felt that on post-treatment scans more focused ^18^F-FDG avidity instead of linear uptake may be indicative of a poor response. Unfortunately, other modalities that have been extensively studied for predicting pathologic response – including endoscopic biopsy, endoscopic ultrasonography, and CT – yielded unsatisfactory results [38, 48]. Recently, diffusion-weighted magnetic resonance imaging has been suggested as potentially powerful tool for this purpose [49], but this tool has not yet been described in the setting of a three-step treatment strategy and requires further validation.

Besides pathologic response, ^18^F-FDG PET response (∆TLG) after induction chemotherapy was also significantly associated with progression-free survival (*p* = 0.02) – but not with overall survival (*p* = 0.18) – in the current study. This finding is supported by a previous prospective study in which ^18^F-FDG PET responders to induction chemotherapy had significantly improved progression-free survival (*p* = 0.02), but not overall survival (*p* = 0.29) [24]. In this way, the early response to induction chemotherapy apparently is an indicator of tumour biology and the likelihood of treatment failure. As such, the early ^18^F-FDG PET response after induction chemotherapy could aid in patient selection for treatment intensification or modification aiming to reduce the high risk of locoregional and distant recurrences in the poor responders.

Certain limitations apply to this study. First, the study was retrospective by nature. Second, different regimens of induction chemotherapy and preoperative chemoradiotherapy were applied in this study. However, our analysis was strengthened by including the largest sample size for this topic so far, using a prospectively maintained database, and using modern ^18^F-FDG PET techniques and imaging analysis.

In conclusion, this study demonstrated that ^18^F-FDG PET seems useful to predict a poor pathologic response early after induction chemotherapy in patients with oesophageal adenocarcinoma undergoing a three-step treatment strategy. As such, the early ^18^F-FDG PET response after induction chemotherapy has the potential to aid in individualized treatment decision-making in this group of patients. However, the standard use of ^18^F-FDG PET for this indication cannot yet be recommended, as the findings (e.g., the determined threshold) of the current exploratory study require external validation. Also, a larger sample size is desired as the 95 % CIs of the estimated diagnostic performance indices in the current study were relatively wide. Also, additional studies are required to determine and validate whether ^18^F-FDG PET alone or in combination with other modalities provides sufficient accuracy to justify modification or withdrawal of subsequent CRT prior to surgery.
